# Supplementary material for: Research on grain production efficiency in China’s main grain producing areas from the perspective of financial support
Source: PLoS One. 2021 Mar 15;16(3):e0247610. doi: 10.1371/journal.pone.0247610 (PMC7959381; doi:10.1371/journal.pone.0247610)
Supplement: S1 Table — (DOCX) [file pone.0247610.s001.docx]

**S1 Table. TFP and decomposition index of grain production from 2001 to2017.**

| YEAR | TFP | TECH | EC | BPC | SECH |
| --- | --- | --- | --- | --- | --- |
| 2001/2002 | 0.988 | 1.013 | 0.976 | 0.976 | 1 |
| 2002/2003 | 0.915 | 0.942 | 0.976 | 1.006 | 0.97 |
| 2003/2004 | 1.045 | 0.994 | 1.053 | 1.012 | 1.041 |
| 2004/2005 | 0.989 | 1.015 | 0.974 | 0.986 | 0.988 |
| 2005/2006 | 1.004 | 1.068 | 0.946 | 0.96 | 0.985 |
| 2006/2007 | 0.979 | 0.911 | 1.075 | 1.028 | 1.046 |
| 2007/2008 | 1.024 | 1.11 | 0.925 | 0.973 | 0.951 |
| 2008/2009 | 0.969 | 0.907 | 1.071 | 1.028 | 1.042 |
| 2009/2010 | 0.945 | 1.054 | 0.840 | 0.906 | 0.927 |
| 2010/2011 | 1.023 | 1.003 | 1.022 | 0.98 | 1.043 |
| 2011/2012 | 1.005 | 1.049 | 0.969 | 0.961 | 1.008 |
| 2012/2013 | 1.014 | 1.031 | 0.991 | 1.02 | 0.972 |
| 2013/2014 | 0.985 | 0.962 | 1.031 | 0.984 | 1.048 |
| 2014/2015 | 1.018 | 1.016 | 1.008 | 1.025 | 0.983 |
| 2015/2016 | 1.013 | 1.037 | 0.983 | 0.967 | 1.017 |
| 2016/2017 | 1.011 | 1.003 | 1.009 | 1.019 | 0.99 |
| MEAN | 0.995 | 1.007 | 0.991 | 0.989 | 1.001 |
