# Supplementary material for: Research on grain production efficiency in China’s main grain producing areas from the perspective of financial support
Source: PLoS One. 2021 Mar 15;16(3):e0247610. doi: 10.1371/journal.pone.0247610 (PMC7959381; doi:10.1371/journal.pone.0247610)
Supplement: Attachment — Submitted filename: Review Report (PONE-D-20-38013).pdf [file pone.0247610.s002.pdf]

Review Report

Manuscript ID: PONE-D-20-38013

Manuscript Title: **Research on grain production efficiency in China's main grain producing areas from the perspective of financial support**

Using the DEA global Malmquist productivity index model, the paper assesses temporal and spatial differences of grain production efficiency in 13 major grain-producing areas in China. To do so, the paper employs a longitudinal provincial-level dataset with data from 2008-2017. The findings suggest an overall reduction in grain production over the period of 2001 to 2017, with an average annual decline of 0.6% mainly due to technical inefficacies in production. The findings also suggested unbalanced spatial development in grain production across the major grain-producing areas. In light of the empirical results, the study is concluded by providing a number of policy recommendations.

Overall, the manuscript good insights into China's grain production sector, however there are some issues that need to be addressed.

1. Major language issues: The manuscript suffers major language and grammatical problems including improper choice of words, punctations, spelling mistakes, grammatical errors, ambagious and confusing sentences, etc. It does not look like an academic article, as the authors use colloquial language. The manuscript needs to undergo a professional language proof reading stage before publication. Let us give a couple of examples:
   (i) Line 44-54: in line 46, the author begins the sentence by "however…" why "however"? The sentence that follows is not a contradiction of the previous sentence. Same in line 49, the sentence starting with "therefore…", why therefore?. In line 52. What is "mu", it needs to be spelled out. In line 54, "the overall food supply will be severely tested", what does this mean?
   (ii) Lines 116-138: very poor language. I can't understand what the authors are trying to communicate.
   (iii) Line 139: it reads "in order to make up for the defect of existing research". Improper language, the existing research is not defect! Line 140, it reads "firstly, on the research angle of view", I don't understand what this means.
   (iv) Line 140-146: long and confusing sentence with poor and confusing language
   (v) Lines 150-151: the article reads "compared with previous research data, the time span is longer and the year is newer". This does not make any sense. I think the author are trying to say: "in comparison to the previous literature, we use recent data with longer time dimension".
   (vi) Line 159-163. "Kth DMU, f inputs, the input variable is denoted as Xik… g output…….". This should be k^{th}, f , X_{ik}, g, etc. Proper subscripts and superscripts should be used, equations and parts of the equation should be in italic. Where is the consistency?? What is DMU? Spell out for the first time. Decision-making unit. Same goes for lines 191-193.
   (vii) Line 185: "that's going to be…" should be "that is…"

(viii) Line 258-275: Bad English. In Line 259 "…arising from peasant household agriculture production". I don't understand this. Line 262-267, does not relate there, "… we should put an end to the phenomenon of arbitrary occupation…", the authors give recommendations here under the "empirical analysis section"
(ix) Line 270: what is armers?
(x) Line 295: "… have not improved their own agriculture production methods". Firstly, bad English. Secondly, based on what evidence the authors claim this? Provide a citation and explain why.
(xi) Line 300: "…productivity index of these six provinces is not high.", what does "not high" imply? what is the limit of high? Bad English. Same mistake in line 254, what is "relatively not high"?
(xii) Line 318: what is ain?
(xiii) Line 323, and 340: Punctations issues: put comma before the word "respectively"
(xiv) Line 338: "thus showing the low performance of low input and low output in grain production". Ambiguous and confusing. I can't understand this.
(xv) Line 368, "disconnect" should be "disconnection"

These are only a few examples that I wanted to point out to. The entire manuscript has English language problems.

2. The manuscript is poorly structured. Long and confusing sentences, paragraphs , and sections, lack of sub-headings etc.

(i) Line 80-84: While, the authors discusses the presentation of the paper: "the second part introduces….. the third part introduces…", the "parts" are unowned and unnumbered. As a reader I do not understand which is part 4 and which is part 2. Also they are not parts, they are sections.
(ii) Under the sub-heading in line 294 "time variation characteristics of TPF and its deco composition index of grain production in main grain producing areas", the section is nearly 9 pages long! This too long and confusing for the readers, as one could easily lose focus. Also, might I suggest using shorter sub-headings. The sub-heading in line 245 is extremely long, for instance you don't need to write "Main grain producing areas", the article makes that clear in the previous sections.
(iii) The sub-section in 423 is redundant and repetitive. This section repeats the discussion presented in lines 279 – 300". The two sections disusing geographical/spatial dimensions of grain productivity should be combined. This makes the manuscript unnecessarily too long and repetitive.
(iv) Another example of poor structuring: line 263, the authors provide recommendations under the "empirical strategies section"
(v) Stay consistent in using abbreviations throughout the article. In line 249 the abbreviation of TFP is used, it is never spelled out in earlier pages. Once TFP is used, continue using TPF in the following pages, instead of "Total Factor Productivity" for maintaining consistency.
(vi) No page numbers.

3. The introduction section is not clear and does not outline the problem, scope of the problem, research objective and questions. It also lacks coherency. The authors provide present in different scales, making it hard to compare. For instance, the aggregate grain output 617.91 tons, but consumption is 479 kg per person. It would be better to calculate production per capita, so one could compare it with per capita consumption.
4. The literature review discusses different estimation approaches but does not provide justification for the preferences of the DEA method. Why is DEA the best method? For instance, in line 164, the approach assumes CRS. In reality, the CRS property of the production function may not hold. Moreover, the DEA does not allow to separate random error from inefficacy, etc. see for example
   Wadud, A., White, B., 2000. Farm household efficiency in Bangladesh: a comparison of stochastic frontier and DEA methods. Applied Economics. 32, 1665–1673
5. The other limitation comes from data. Using provincial level data does not capture micro- or farm-level variations. Total population of a province is used as labour input in production. The findings are indicative at best. Hence the authors need to clearly write limitations of the study, especially limitations attributable to data. This is the major empirical short-coming in the paper, so it needs to stand out.
6. Line 282 and 293. The total factor productivity index greater and smaller than 1. The author needs to clearly define and interpret the index, discuss the min and max range of the index and what they signify. Does higher index signify more productivity? Why the value 1 is regarded as a benchmark? Discuss and clarify to help the reader better understand the estimated ranges of the index.
